# Albumin–Bilirubin Score Differentiates Liver Fibrosis Stage and Hepatocellular Carcinoma Incidence in Chronic Hepatitis B Virus Infection: A Retrospective Cohort Study

**DOI:** 10.4269/ajtmh.19-0129

**Published:** 2019-05-20

**Authors:** Koji Fujita, Takako Nomura, Asahiro Morishita, Kyoko Oura, Hirohito Yoneyama, Hideki Kobara, Kunihiko Tsutsui, Takashi Himoto, Tsutomu Masaki

**Affiliations:** 1 Department of Gastroenterology and Neurology, Faculty of Medicine, Kagawa University, Takamatsu, Japan;; 2 Department of Medical Technology, Kagawa Prefectural University of Health Sciences, Takamatsu, Japan

## Abstract

The albumin–bilirubin (ALBI) score was originally established to stratify prognosis in patients with cirrhosis. The diagnostic accuracy of ALBI score in liver fibrosis staging in patients with chronic hepatitis B remains to be investigated. The present retrospective study, therefore, aimed to evaluate the ability of this score to stage liver fibrosis in these patients. Briefly, consecutive patients with hepatitis B virus (HBV) infection who underwent liver biopsy examinations in Kagawa University Hospital were enrolled. Liver fibrosis stage was assessed using a modified Meta-Analysis of Histological Data in Viral Hepatitis score. Albumin–bilirubin scores were calculated according to the following equation: (log_10_ total bilirubin [T-Bil] × 0.66) + (albumin [Alb] × −0.085). A total of 91 patients were enrolled in this study. Albumin–bilirubin score was able to differentiate stage 4 from stage 3 fibrosis (*P* < 0.05). When an ALBI score of −2.190 was adopted as the cutoff value for differentiating stage 4 from stages 1–3, the sensitivity, specificity, and positive likelihood ratio were 85.7%, 74.0%, and 3.300, respectively. Kaplan–Meier analysis showed that baseline ALBI scores < −2.190 correlated with better hepatocellular carcinoma (HCC)–free survival (*P* < 0.05). In conclusion, ALBI score can be used for liver fibrosis staging in Japanese chronic hepatitis B patients and can help distinguish cirrhotic from non-cirrhotic status. Furthermore, ALBI score was useful as a prognosis biomarker in our patients, with smaller ALBI scores predicting better HCC-free survival. Because calculating ALBI score is easy using serum T-Bil and Alb alone, ALBI score will help clinicians with decision-making in management of HBV-infected patients.

## INTRODUCTION

Hepatitis B virus (HBV) infection is a major public health problem worldwide. When defined as hepatitis B surface (HBs) antigen positivity, HBV infection is estimated to affect 257 million living people, resulting in 887,000 deaths in 2015.^1^ If left untreated, chronic hepatitis B progresses to liver cirrhosis in up to 40% of patients.^2,3^ According to an observational study, approximately 30% of cirrhotic patients then develop hepatocellular carcinoma (HCC) in 10 years.^4^ As such, evaluating the degree of fibrosis is inevitable in the management of patients with HBV infection.^5^

Management and treatment of HBV infection in low-income and middle-income countries differs substantially from that in developed countries because of the difficulty in accessing medical resources.^6^ Measuring serum direct biomarkers, such as *Wisteria floribunda* agglutinin-positive Mac-2 binding protein (WFA-M2BP) and Enhanced Liver Fibrosis score, and performing liver biopsy examination for patients with HBV infection would mean great economic burden in limited health-care budgets.^7,8^ Simpler and less-expensive methods to evaluate liver fibrosis stage is to be established.

The albumin–bilirubin (ALBI) score was originally developed to predict prognosis in patients suffering from liver cirrhosis with or without HCC.^9^ According to this score, which is calculated based on serum total bilirubin (T-Bil) and albumin (Alb) alone, patient prognosis is classified into one of three grades, called the ALBI grade. However, the diagnostic accuracy of the ALBI score when used for liver fibrosis staging in patients with chronic hepatitis has not been investigated well. The purpose of this study was, therefore, to evaluate the ability of the ALBI score to stage liver fibrosis in Japanese HBV-infected patients with chronic hepatitis and cirrhosis.

## MATERIALS AND METHODS

### Ethics.

This study was conducted in accordance with the ethical principles of the Declaration of Helsinki and was approved by the Institutional Review Board of Kagawa University, Faculty of Medicine (Heisei-30-151).^10^ Informed consent was obtained for the analysis of clinical data for the present study. For patients who died and had no relatives listed in their clinical records, we provided opt-out methods for their relatives by publishing a summary of this study on our university website.^11,12^

### Patients.

Consecutive patients with HBV infection who underwent percutaneous liver biopsy examinations in a clinical setting between November 1, 1986 and October 31, 1999, in Kagawa University Hospital were retrospectively enrolled. Hepatitis B virus infection was defined as positivity for HBs antigen, HBs antibody, hepatitis B core antibody, or DNA polymerase, as described in patient medical records. Patients were excluded if they had HCC at the time of liver biopsy examination.

### Clinical data.

The following clinical data were extracted from the participants’ medical records: age, gender, platelet count, aspartate aminotransferase (AST) level, alanine aminotransferase (ALT) level, T-Bil, and serum Alb. Total bilirubin (mg/dL) was converted to T-Bil (µmol/L) according to the following equation: T-Bil (mg/dL) × 17.2. Albumin (g/dL) was converted to Alb (g/L) according to the following equation: Alb (g/dL) × 10. Albumin–bilirubin scores were calculated according to the equation defined in the original report: (log_10_T-Bil [µmol/L] × 0.66) + (Alb [g/L] × −0.085).^9^

### Histopathological analysis.

The extent of fibrosis was assessed using a modified Meta-Analysis of Histological Data in Viral Hepatitis (METAVIR) score. Stages of fibrosis were defined as follows: stage 1, portal or central fibrosis; stage 2, some septa; stage 3, many septa; and stage 4, cirrhosis.^13^ The METAVIR grading system was used to assess hepatic inflammatory activity in patients with fibrosis stages 1–3.^14^ Staging and grading were conducted by an experienced pathologist who specialized in liver pathology.

### Statistical analysis.

Continuous data, presented as the median and interquartile range (IQR), were analyzed using the Mann–Whitney *U* test or Wilcoxon matched-pairs signed rank test. Kaplan–Meier curves were analyzed using the log-rank (Mantel–Cox) test. *P*-values less than 0.05 were regarded statistically significant. Analyses were performed using GraphPad Prism 5 (GraphPad Software, La Jolla, CA).

## RESULTS

### Patient characteristics.

A total of 91 patients comprising 51 males and 40 females were enrolled in this study. Based on liver biopsy examinations, 27 patients had stage 1 fibrosis, 24 had stage 2 fibrosis, 26 had stage 3 fibrosis, and 14 had stage 4 fibrosis. Among the 91 patients, 57 were followed up for 1 year or more after the initial liver biopsy. The longest follow-up period was 32 years. Hepatocellular carcinoma was diagnosed in 13 patients. Other clinical data including age, platelet count, Alb, AST, ALT, and T-Bil are presented in Table 1.

**Table 1:** Patients’ characteristics

Fibrosis stage	Total	F1	F2	F3	F4
Patient number	91	27	24	26	14
Age	40 (30–55)	46 (35–59)	48 (40–58)	54 (45–60)	54 (41–64)
Gender (male/female)	54/40	15/12	11/13	17/9	8/6
Albumin (g/dL)	3.7 (3.4–4.0)	3.7 (3.4–4.0)	3.9 (3.6–4.2)	3.7 (3.5–3.9)	3.1 (2.7–3.5)
AST (U/L)	71 (41–110)	58 (32–84)	64 (44–107)	97 (45–127)	59 (41–114)
ALT (U/L)	92 (49–142)	96 (53–130)	92 (50–178)	115 (58–209)	40 (30–78)
Total bilirubin (mg/dL)	0.9 (0.7–1.3)	0.8 (0.6–1.2)	0.9 (0.6–1.1)	1.1 (0.7–1.3)	1.3 (0.9–2.0)
Follow-up < 1 year patient number	34	11	6	12	5
Follow-up > 1 year patient number	57	16	18	14	9
Year	13.0 (10.0–22.5)	9.0 (13.5–25.0)	17.0 (10.0–28.0)	10.0 (14.0–21.0)	11.0 (8.5–14.5)
HCC prevalence patient number	13	2	5	4	2

ALT = alanine aminotransferase; AST = aspartate aminotransferase level; HCC = hepatocellular carcinoma. Continuous data are presented as the median and interquartile range.

### Diagnostic ability of the ALBI score in liver fibrosis staging.

**Figure 1.**
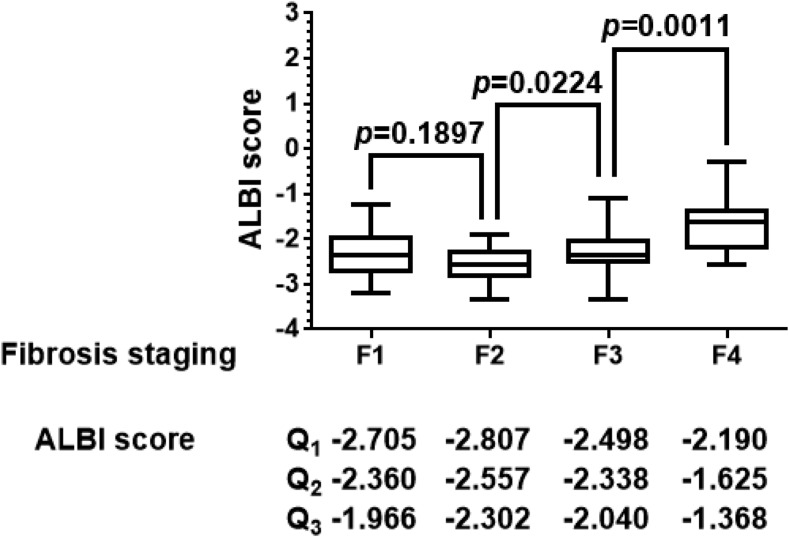
Albumin–bilirubin (ALBI) score differentiated cirrhosis from non-cirrhotic status. Differences in the median ALBI score between two fibrosis stages were analyzed using the Mann–Whitney *U* test. Albumin–bilirubin scores significantly differed between stage 3 and stage 2 fibrosis and between stage 4 and stage 3 fibrosis (*P* < 0.05). However, median ALBI scores did not significantly differ between stages 2 and 1 (*P* > 0.05). *P* values less than 0.05 were considered statistically significant. Q_1_ = 25th percentile value; Q_2_ = median value; Q_3_ = 75th percentile value.

Differences in the median ALBI score between two fibrosis stages were analyzed using the Mann–Whitney *U* test (Figure 1). We found that ALBI scores significantly differed between stage 3 and stage 2 fibrosis and between stage 4 and stage 3 fibrosis (*P* < 0.05). However, median ALBI scores did not significantly differ between stages 2 and 1 (*P* > 0.05).

**Figure 2.**
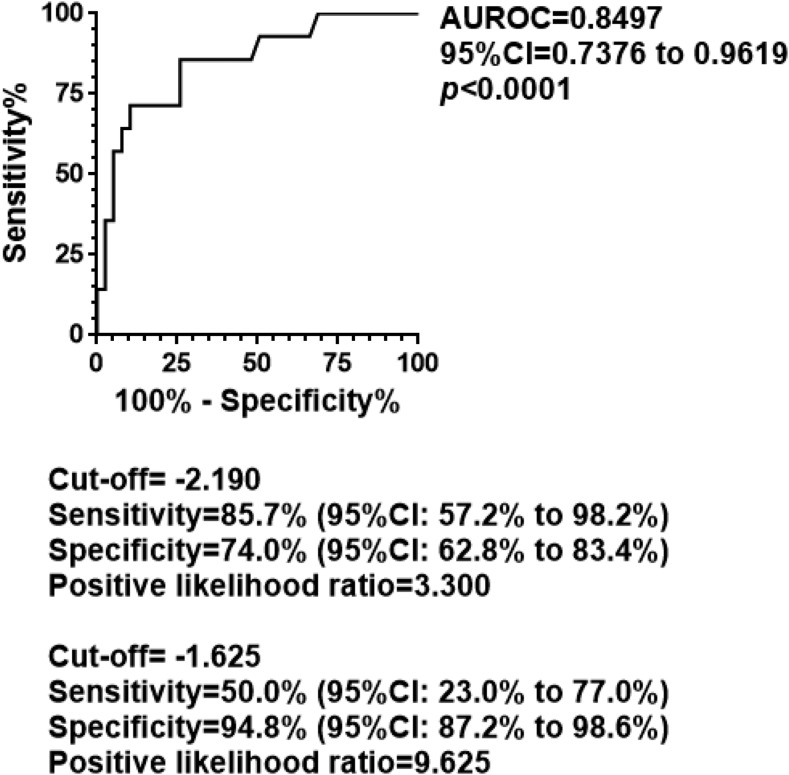
Diagnostic ability of the albumin–bilirubin (ALBI) score in liver fibrosis staging. Receiver operating characteristic (ROC) analysis was also performed to assess the ability of the ALBI score to distinguish cirrhotic (stage 4) from non-cirrhotic (stages 1–3) status. The area under the ROC curve was 0.8497. When an ALBI score of −2.190, the 25th percentile value of stage 4 patients, was adopted as the cutoff value for differentiating cirrhotic from non-cirrhotic status, sensitivity and specificity were 85.7% and 74.0%, respectively, with a positive likelihood ratio of 3.300. When the cutoff value was instead set at −1.625, the median ALBI score of stage 4 patients, specificity and the positive likelihood ratio improved to 94.8% and 9.625, respectively, whereas sensitivity decreased to 50.0%. *P*-values less than 0.05 were considered statistically significant.

Receiver operating characteristic (ROC) analysis was also performed to assess the ability of the ALBI score to distinguish cirrhotic (stage 4) from non-cirrhotic (stages 1–3) status. As shown in Figure 2, the area under the ROC curve (AUROC) was 0.8497. When an ALBI score of −2.190, the 25th percentile value of stage 4 patients, was adopted as the cutoff value for differentiating cirrhotic from non-cirrhotic status, sensitivity and specificity were 85.7% and 74.0%, respectively, with a positive likelihood ratio of 3.300 (Figure 2). When the cutoff value was instead set at −1.625, the median ALBI score of stage 4 patients, specificity and the positive likelihood ratio improved to 94.8% and 9.625, respectively, whereas sensitivity decreased to 50.0%.

### Hepatitis activity modifies ALBI score.

**Figure 3.**
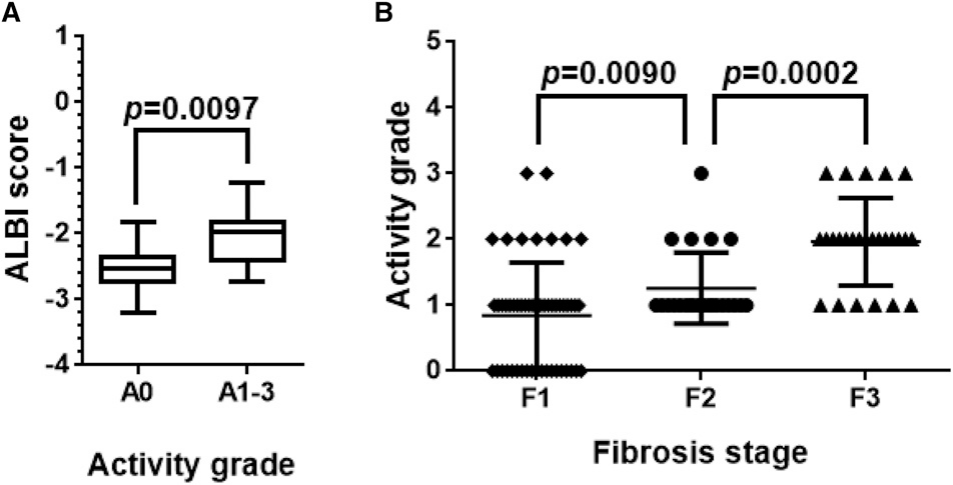
Hepatitis activity modifies ALBI score. The patients with stage 1 fibrosis were classified according to hepatitis activity as follows: activity grade 0, 18 patients; activity grade 1, 5 patients; activity grade 2, 3 patients; activity grade 3, 1 patient (based on a modified METAVIR score). (**A**) The ALBI scores for the 18 patients with activity grade 0 were significantly different from those for the 9 patients with grades 1--3. (**B**) Activity grade was greater in fibrosis stage 2 patients than stage 1, and in stage 3 patients than stage 2. The Mann-Whitney U test was applied for statistical analysis, and *P* values less than 0.05 were considered statistically significant.

To assess fluctuation of ALBI score depending on histopathological activity grade, 27 patients with fibrosis stage 1 were classified as follows: activity grade 0, 18 patients; activity grade 1, five patients; activity grade 2, three patients; and activity grade 3, one patient. As shown in Figure 3A, the ALBI scores for the 18 patients with grade 0 activity were significantly different from those for the nine patients with grade 1–3 activity using the Mann–Whitney *U* test (*P* < 0.05). Activity grade was greater in fibrosis stage 2 patients than in stage 1 and in stage 3 patients than in stage 2, as shown in Figure 3B.

### Hepatocellular carcinoma incidence and overall survival.

In the present cohort, 57 patients were followed up for 1 year or more. Among these patients, 13 were diagnosed with HCC during follow-up. The median period between initial liver biopsy and HCC diagnosis was 9.0 years (IQR: 7.5–13.0 years).

**Figure 4.**
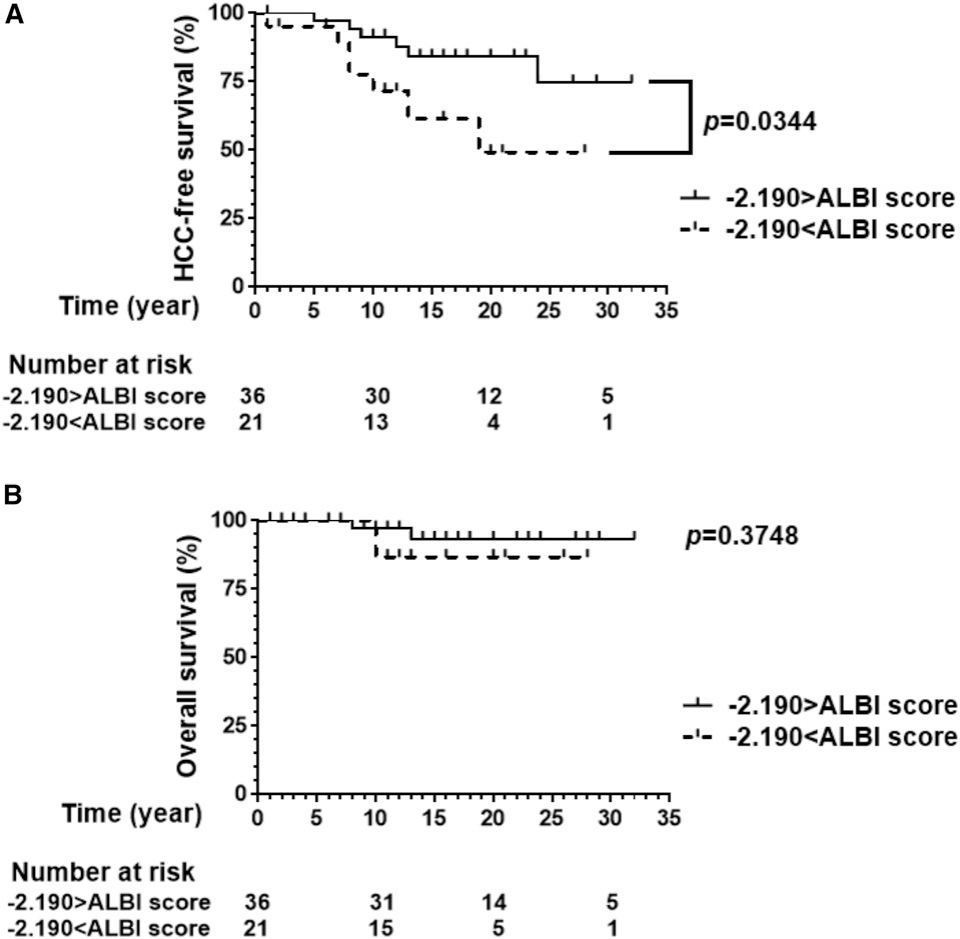
Hepatocellular carcinoma (HCC) incidence and overall survival. In the present cohort, 57 patients were followed up for 1 year or more. Using a baseline albumin–bilirubin (ALBI) score of −2.190 as a cutoff value, the 57 patients were divided into two groups: the smaller ALBI score group with scores < −2.190, and the greater ALBI score group with scores > −2.190. (**A**) Kaplan–Meier analysis revealed that HCC-free survival was significantly poorer in the greater ALBI score group than the lower (*P* < 0.05). (**B**) For overall survival, smaller ALBI scores did not correlate with better survival (*P* > 0.05). Kaplan–Meier curves were analyzed using log-rank (Mantel–Cox) test. *P*-values less than 0.05 were considered statistically significant.

Using a baseline ALBI score of −2.190 as a cutoff value, the 57 patients were divided into two groups: the smaller ALBI score group with scores < −2.190 and the greater ALBI score group with scores > −2.190. Kaplan–Meier analysis revealed that HCC-free survival was significantly poorer in the greater ALBI score group than the smaller (*P* < 0.05; Figure 4A). For overall survival, smaller ALBI scores did not correlate with better survival (Figure 4B). Limiting to 13 patients who were diagnosed with HCC, patients’ status at the last follow-up was reviewed. As shown in Table 2, 50% of patients were cured of HCC at the last follow-up in the smaller ALBI score group, whereas as little as 14% of patients completed curative therapy of HCC in the greater ALBI score group.

**Table 2:** Status at the last follow-up of patients who developed HCC during the follow-up period

	Total	Smaller ALBI score	Greater ALBI score
Patient number (%)	13	6 (100)	7 (100)
Status at the last follow-up			
Death	3	1 (17)	2 (29)
Alive with HCC complication	4	2 (33)	4 (57)
Alive with HCC-free status	6	3 (50)	1 (14)

ALBI = albumin–bilirubin; HCC = hepatocellular carcinoma.

### Changes in ALBI scores during follow-up.

**Figure 5.**
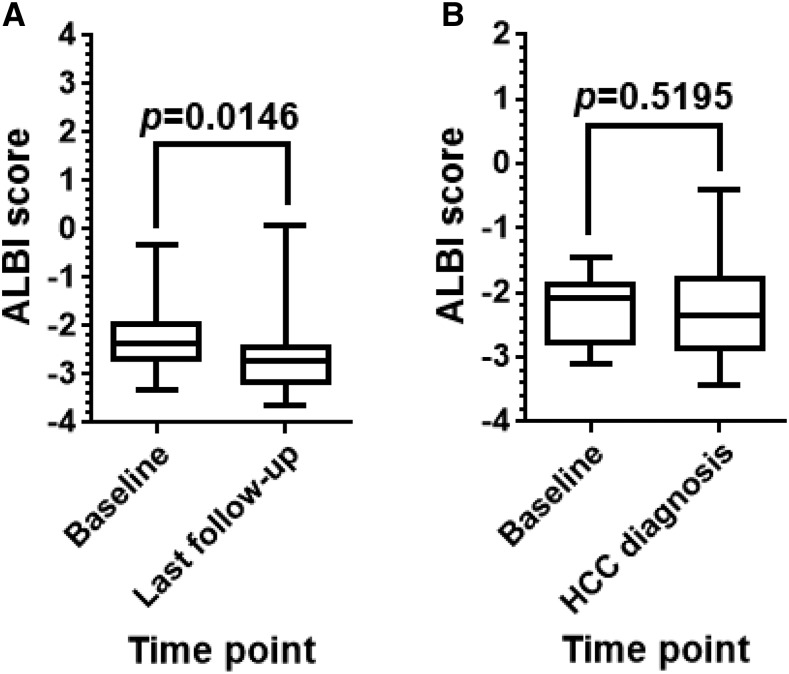
Changes in albumin–bilirubin (ALBI) scores during follow-up. In the present cohort, 78 patients remained hepatocellular carcinoma (HCC)–free during follow-up, whereas the other 13 patients were diagnosed with HCC. (**A**) In the HCC-free patients, ALBI scores were significantly better at the end of follow-up (last follow-up) than at baseline when the initial liver biopsy was conducted (*P* < 0.05). (**B**) In the HCC-diagnosed patients, however, ALBI scores at HCC diagnosis did not significantly differ from those at baseline (*P* < 0.05). Data were analyzed using Wilcoxon matched-pairs signed rank test. *P*-values less than 0.05 were considered statistically significant.

In the present cohort, 78 patients remained HCC-free during follow-up, whereas the other 13 patients were diagnosed with HCC. In the HCC-free patients, ALBI scores were significantly better at the end of follow-up (last follow-up) than at baseline when the initial liver biopsy was conducted (Figure 5A). In the HCC-diagnosed patients, however, ALBI scores at HCC diagnosis did not significantly differ from those at baseline (Figure 5B).

## DISCUSSION

The present study has two main findings. First, ALBI scores significantly differed between fibrosis stages, indicating that the ALBI score can be used for fibrosis staging. In particular, it can be used to distinguish between advanced liver fibrosis and cirrhosis. Second, smaller ALBI scores correlated with better HCC-free survival, suggesting that the ALBI score may be useful in predicting patient prognosis. Together, these findings suggest that application of the ALBI score can be expanded from cirrhosis alone to chronic liver diseases, including chronic hepatitis B.

The ALBI score was originally established to classify patients with cirrhosis according to their prognosis.^9^ To be specific to the etiology of HBV, the ALBI score predicts prognosis in HBV-related cirrhosis and HCC,^15,16^ with higher ALBI scores at admission being predictive of a higher 3-month mortality rate in patients with HBV-related acute-on-chronic liver failure.^17^ The ALBI score also retains prognostic value in HBV-related HCC treated with transarterial chemoembolization therapy.^18^ In general, ALBI scores can be classified as follows: < −2.600, better prognosis, grade 1; −2.600 to −1.390, moderate prognosis, grade 2; and > −1.390, worse prognosis, grade 3. At present, application of the ALBI score has been limited to patients with cirrhosis, similarly to the Child–Pugh and Model for End-Stage Liver Disease scores.^19,20^

The present study proposes a cutoff value of −2.190 as a diagnostic threshold for HBV-related cirrhosis. Patients with an ALBI score > −2.190 are classified as having ALBI grade 2 and 3 cirrhosis, consistent with the conventional cutoff values. However, patients with an ALBI score < −2.190 are classified as non-cirrhotic in our study, whereas they would be classified as cirrhotic (grade 1 or 2) using the conventional cutoff values. We speculate that the discrepancy might be partially due to how cirrhosis was defined in the cohorts on which the conventional cutoff values were based and that cirrhosis might be clinically diagnosed in a proportion of patients, not histopathologically.

In the present study, the ALBI score was shown to have potential in being applied for liver fibrosis staging and stratifying prognosis in patients with HBV infection, including chronic hepatitis cases. As a biomarker of liver fibrosis stage, we found that the ALBI score has a moderate diagnostic ability, distinguishing cirrhotic from non-cirrhotic status with an AUROC of between 0.8 and 0.9. This may be superior to the conventional indirect biomarker, AST to Platelet Ratio Index (APRI).^21^ Furthermore, when we increased the cutoff ALBI score for cirrhosis, the ALBI score could diagnose cirrhosis with more than 90% specificity, a level comparable with that of the Fibrosis-4 (FIB-4) index.^2^ Albumin–bilirubin score is also able to compete with an emerging direct biomarker, WFA-M2BP,^22^ which has an AUROC of 0.689 when used to differentiate cirrhotic from non-cirrhotic status.^7^ Thus, the ALBI score appears to diagnose cirrhosis with an accuracy comparable with that of the current indirect and direct biomarkers.

In a cohort of 382 patients with hepatitis C virus (HCV) infection, we reported a cutoff value of −2.125 to differentiate cirrhotic from non-cirrhotic status with an AUROC of 0.815, sensitivity of 73.2%, specificity of 87.1%, and positive likelihood ratio of 5.67.^23^ In the present study, the closest value to −2.125 was −2.121 with a sensitivity of 66.67%, specificity of 72.34%, and positive likelihood ratio of 2.410, as shown in Supplemental Table 1. In the perspective of the AUROC, the diagnostic accuracy of ALBI score for HBV-related cirrhosis might be lower than that for HCV-related cirrhosis partially because the cohort in the present study is smaller than the one in the past report.

Values of fibrosis biomarkers vary with hepatitis activity grade.^24^ The ALBI score also fluctuated according to activity grade in fibrosis stage 1 patients in the present study. However, the median ALBI scores of fibrosis stages 1 and 2 were not statistically different, although activity grades were greater in stage 2 than in stage 1. Thus, activity grade might have a minor influence on ALBI score compared with fibrosis stage.

Regarding our investigation of the ALBI score as a prognosis biomarker in patients with HBV infection, we found that lower baseline ALBI scores correlated with better HCC-free survival. This suggests that smaller ALBI scores at baseline predict better HCC-free survival. In terms of overall survival, ALBI score did not differentiate patients’ prognosis in the cohort. However, a larger proportion of patients in the higher ALBI score group still carried HCC at the last follow-up than in the lower ALBI score group. If observations were not censored, the difference in overall survival rates between the two groups would probably be greater because 4-year survival rates in HBV-related HCC patients were reported to be less than 10% at that time.^25^

We also found that ALBI scores were unchanged over time in patients later diagnosed with HCC, indicating that lack of ALBI score improvement may predict HCC occurrence. By contrast, ALBI scores at the last follow-up were improved compared with baseline in patients free from HCC through their observation periods. This finding may be partially attributable to the oldest antiviral agent, lamivudine. Lamivudine was covered under public insurance beginning in November 2000 in Japan and might partially improve hepatic inflammation and fibrosis. Together, these data suggest that application of the ALBI score should not be limited to liver cirrhosis as it is able to stratify prognosis in HBV-infected patients both with and without cirrhosis.

The ALBI score differs from other fibrosis indices, such as FIB-4 index and APRI, in that it is calculated without platelet count. This means that the diagnostic accuracy of the ALBI score in liver fibrosis staging is retained in diseases that are accompanied by thrombocytopenia or thrombocythemia. In daily practice, these may include idiopathic or drug-induced thrombocytopenia, *Helicobacter pylori* infection, and iron deficiency anemia, all of which are frequently observed. In patients unaffected by such diseases, platelet-ALBI grade, which was recently introduced for patients with HCC and incorporates platelet count into the ALBI score,^26,27^ may be used instead. It is possible that combining platelet count with ALBI measurement would allow more accurate determination of fibrosis stage in patients with chronic hepatitis, although this requires further investigation.

Despite the insights provided by this study, there are some limitations to consider. First, the case number was relatively small. Second, HBV genotypes and viral doses in sera were not determined in most of the patients. Third, the diagnostic accuracy of ALBI score for cirrhosis was not compared with that of shear wave elastography or magnetic resonance elastography.^28,29^ The current data need to be validated in a greater cohort with more detailed information of patients with HBV infection.

In conclusion, the ALBI score can be used to stage liver fibrosis in patients with HBV infection. In particular, the ALBI score can be used to differentiate cirrhotic from non-cirrhotic status with an accuracy that is comparable with that of other serum biomarkers. Furthermore, smaller ALBI scores predicted better HCC-free survival. Together, these results demonstrated that clinical application of the ALBI score can be expanded from cirrhosis alone to chronic liver diseases, including chronic hepatitis B.

## Supplementary Files

Supplemental table
