# Supplementary material for: Albumin–Bilirubin Score Differentiates Liver Fibrosis Stage and Hepatocellular Carcinoma Incidence in Chronic Hepatitis B Virus Infection: A Retrospective Cohort Study
Source: Am J Trop Med Hyg. 2019 May 20;101(1):220–5. doi: 10.4269/ajtmh.19-0129 (PMC6609180; doi:10.4269/ajtmh.19-0129)
Supplement: Supplementary file 1 [file tpmd190129.SD1.pdf]

Supplementary table 1

Cut-off values to differentiate cirrhosis from noncirrhotic status in HBV infection using ALBI score

Cut-off value	Sensitivity (%)	95% Confidence interval	Specificity (%)	95% Confidence interval	Positive likelihood ratio
-3.327	100.0	81.47% to 100.0%	1.064	0.02693% to 5.785%	1.011
-3.282	100.0	81.47% to 100.0%	2.128	0.2587% to 7.475%	1.022
-3.223	100.0	81.47% to 100.0%	3.191	0.6631% to 9.045%	1.033
-3.182	100.0	81.47% to 100.0%	4.255	1.171% to 10.54%	1.044
-3.128	100.0	81.47% to 100.0%	5.319	1.749% to 11.98%	1.056
-3.070	100.0	81.47% to 100.0%	6.383	2.378% to 13.38%	1.068
-3.039	100.0	81.47% to 100.0%	7.447	3.046% to 14.74%	1.080
-3.028	100.0	81.47% to 100.0%	8.511	3.746% to 16.08%	1.093
-3.013	100.0	81.47% to 100.0%	9.574	4.472% to 17.40%	1.106
-2.998	100.0	81.47% to 100.0%	10.64	5.221% to 18.70%	1.119
-2.970	100.0	81.47% to 100.0%	11.70	5.989% to 19.97%	1.133
-2.927	100.0	81.47% to 100.0%	13.83	7.574% to 22.49%	1.160
-2.860	100.0	81.47% to 100.0%	14.89	8.389% to 23.72%	1.175
-2.815	100.0	81.47% to 100.0%	15.96	9.215% to 24.95%	1.190
-2.802	100.0	81.47% to 100.0%	17.02	10.05% to 26.16%	1.205
-2.773	100.0	81.47% to 100.0%	18.09	10.90% to 27.37%	1.221
-2.744	100.0	81.47% to 100.0%	19.15	11.76% to 28.56%	1.237
-2.732	100.0	81.47% to 100.0%	20.21	12.63% to 29.75%	1.253
-2.718	100.0	81.47% to 100.0%	22.34	14.39% to 32.10%	1.288
-2.702	100.0	81.47% to 100.0%	23.40	15.29% to 33.26%	1.306
-2.693	100.0	81.47% to 100.0%	24.47	16.19% to 34.42%	1.324
-2.681	100.0	81.47% to 100.0%	25.53	17.09% to 35.57%	1.343
-2.658	100.0	81.47% to 100.0%	26.6	18.01% to 36.71%	1.362
-2.622	100.0	81.47% to 100.0%	27.66	18.93% to 37.85%	1.382
-2.583	100.0	81.47% to 100.0%	28.72	19.86% to 38.98%	1.403
-2.562	94.44	72.71% to 99.86%	32.98	23.62% to 43.44%	1.409
-2.559	94.44	72.71% to 99.86%	34.04	24.58% to 44.54%	1.432
-2.555	94.44	72.71% to 99.86%	35.11	25.54% to 45.64%	1.455
-2.541	88.89	65.29% to 98.62%	36.17	26.51% to 46.73%	1.393
-2.523	88.89	65.29% to 98.62%	38.30	28.46% to 48.90%	1.441
-2.511	88.89	65.29% to 98.62%	39.36	29.44% to 49.98%	1.466
-2.496	88.89	65.29% to 98.62%	40.43	30.42% to 51.05%	1.492
-2.482	88.89	65.29% to 98.62%	41.49	31.41% to 52.12%	1.519
-2.474	88.89	65.29% to 98.62%	42.55	32.41% to 53.18%	1.547
-2.458	88.89	65.29% to 98.62%	44.68	34.41% to 55.29%	1.607

-2.438	83.33	58.58% to 96.42%	46.81	36.44% to 57.39%	1.567
-2.417	83.33	58.58% to 96.42%	50.00	39.51% to 60.49%	1.667
-2.382	83.33	58.58% to 96.42%	51.06	40.54% to 61.52%	1.703
-2.361	83.33	58.58% to 96.42%	53.19	42.61% to 63.56%	1.780
-2.353	83.33	58.58% to 96.42%	54.26	43.66% to 64.58%	1.822
-2.338	83.33	58.58% to 96.42%	57.45	46.82% to 67.59%	1.958
-2.319	83.33	58.58% to 96.42%	58.51	47.88% to 68.59%	2.009
-2.307	83.33	58.58% to 96.42%	60.64	50.02% to 70.56%	2.117
-2.280	83.33	58.58% to 96.42%	62.77	52.18% to 72.52%	2.238
-2.239	83.33	58.58% to 96.42%	64.89	54.36% to 74.46%	2.374
-2.218	83.33	58.58% to 96.42%	65.96	55.46% to 75.42%	2.448
-2.203	83.33	58.58% to 96.42%	68.09	57.67% to 77.33%	2.611
-2.191	83.33	58.58% to 96.42%	69.15	58.78% to 78.27%	2.701
-2.184	72.22	46.52% to 90.31%	69.15	58.78% to 78.27%	2.341
-2.158	66.67	40.99% to 86.66%	70.21	59.90% to 79.21%	2.238
-2.135	66.67	40.99% to 86.66%	71.28	61.02% to 80.14%	2.321
*****-2.121	66.67	40.99% to 86.66%	72.34	62.15% to 81.07%	2.410
-2.107	66.67	40.99% to 86.66%	73.40	63.29% to 81.99%	2.507
-2.090	66.67	40.99% to 86.66%	74.47	64.43% to 82.91%	2.611
-2.063	66.67	40.99% to 86.66%	76.60	66.74% to 84.71%	2.848
-2.049	66.67	40.99% to 86.66%	77.66	67.90% to 85.61%	2.984
-2.007	66.67	40.99% to 86.66%	78.72	69.07% to 86.49%	3.133
-1.953	66.67	40.99% to 86.66%	79.79	70.25% to 87.37%	3.298
-1.939	61.11	35.75% to 82.70%	79.79	70.25% to 87.37%	3.023
-1.935	61.11	35.75% to 82.70%	81.91	72.63% to 89.10%	3.379
-1.928	61.11	35.75% to 82.70%	82.98	73.84% to 89.95%	3.590
-1.916	61.11	35.75% to 82.70%	84.04	75.05% to 90.78%	3.830
-1.907	61.11	35.75% to 82.70%	86.17	77.51% to 92.43%	4.419
-1.899	44.44	21.53% to 69.24%	86.17	77.51% to 92.43%	3.214
-1.885	44.44	21.53% to 69.24%	88.30	80.03% to 94.01%	3.798
-1.863	44.44	21.53% to 69.24%	89.36	81.30% to 94.78%	4.178
-1.844	44.44	21.53% to 69.24%	90.43	82.60% to 95.53%	4.642
-1.833	38.89	17.30% to 64.25%	90.43	82.60% to 95.53%	4.062
-1.800	38.89	17.30% to 64.25%	91.49	83.92% to 96.25%	4.569
-1.767	38.89	17.30% to 64.25%	92.55	85.26% to 96.95%	5.222
-1.725	38.89	17.30% to 64.25%	93.62	86.62% to 97.62%	6.093
-1.610	33.33	13.34% to 59.01%	93.62	86.62% to 97.62%	5.222
-1.525	27.78	9.695% to 53.48%	93.62	86.62% to 97.62%	4.352
-1.514	27.78	9.695% to 53.48%	95.74	89.46% to 98.83%	6.528
-1.482	27.78	9.695% to 53.48%	96.81	90.96% to 99.34%	8.704
-1.428	22.22	6.409% to 47.64%	96.81	90.96% to 99.34%	6.963
-1.331	16.67	3.579% to 41.42%	96.81	90.96% to 99.34%	5.222

-1.244	11.11	1.375% to 34.71%	96.81	90.96% to 99.34%	3.481
-1.229	11.11	1.375% to 34.71%	97.87	92.52% to 99.74%	5.222
-1.167	11.11	1.375% to 34.71%	98.94	94.21% to 99.97%	10.44
-0.9475	11.11	1.375% to 34.71%	100.0	96.15% to 100.0%	-
-0.5449	5.556	0.1406% to 27.29%	100.0	96.15% to 100.0%	-

***** The cut-off value= -2.121 was the closest to an alternative cut-off value= -2.125 to differentiate cirrhosis from noncirrhotic status in hepatitis C virus infection with sensitivity 73.2%; specificity 87.1%; and positive likelihood ratio 5.67. [23]

HBV, hepatitis B virus; ALBI score, albumin-bilirubin score
